# Toxicity, Antioxidant Activity, and Phytochemicals of Basil (*Ocimum basilicum* L.) Leaves Cultivated in Southern Punjab, Pakistan

**DOI:** 10.3390/foods11091239

**Published:** 2022-04-26

**Authors:** Hafiz Rehan Nadeem, Saeed Akhtar, Piero Sestili, Tariq Ismail, Susanne Neugart, Muhammad Qamar, Tuba Esatbeyoglu

**Affiliations:** 1 Institute of Food Science and Nutrition, Bahauddin Zakariya University, Multan 60800, Pakistan; hrnfoodscientist@gmail.com (H.R.N.); ammarbintariq@yahoo.com (T.I.); muhammad.qamar44@gmail.com (M.Q.); 2 Department of Biomolecular Sciences, Università degli Studi di Urbino Carlo Bo, 61029 Urbino, Italy; piero.sestili@unirub.it; 3 Department of Crop Sciences, Division Quality and Sensory of Plant Products, Georg-August-Universität Göttingen, Carl-Sprengel-Weg 1, 37075 Goettingen, Germany; susanne.neugart@uni-goettingen.de; 4 Institute of Food Science and Human Nutrition, Department of Food Development and Food Quality, Gottfried Wilhelm Leibniz University Hannover, Am Kleinen Felde 30, 30167 Hannover, Germany

**Keywords:** *Artemia salina*, brine shrimp, coumarin, DPPH, FRAP, mass spectrometry, polyphenol, radical scavenging

## Abstract

Basil *(Ocimum basilicum* L.) is one of the most common aromatic herbs, a rich source of bioactive compounds, and is used extensively to add aroma and flavor to food. The leaves, both in fresh and dried form, are used as a culinary ingredient in different cultures. *O. basilicum* is also famous for its therapeutic potential and preservation effects. The present study investigated the cytotoxicity of basil at three different growth stages (GS), i.e., GS-1 (58 days of growth), GS-2 (69 days of growth), and GS-3 (93 days of growth) using the brine shrimp lethality assay. The results revealed that cytotoxicity was influenced by GS and the concentration of extracts. Aqueous extracts of basil at a concentration of 10 to 1000 µg/mL did not show notable toxicity. The lowest mortality rate, i.e., 8.9%, was recorded for GS-2 at the highest tested dose of basil extracts. The mortality rate at GS-1, GS-2, and GS-3 was found to be 26.7 ± 3.34%, 8.91 ± 0.10%, and 16.7 ± 0.34%, respectively, at 1000 µg/mL. GS-2 basil powder with the lowest toxicological risk was extracted with different solvents, viz., *n*-hexane, dichloromethane, ethanol, and water. The highest concentration of plant secondary metabolites including total phenolic acid, flavonoids, and tannin content was observed in ethanol extracts. Ethanol extracts also exhibited the highest antioxidant activity in DPPH, FRAP and H_2_O_2_ assays. LC-ESI-MS/MS analysis presented ethanol extracts of basil as a promising source of known health-promoting and therapeutic compounds such as rosmarinic acid, ellagic acid, catechin, liquiritigenin, and umbelliferone. The results suggest basil, a culinary ingredient, as a potential source of bioactive compounds which may offer an array of health promoting and therapeutic properties.

## 1. Introduction

Bioactive compounds of plant origin are well appreciated for their extended applications in food preservation, value addition, and as therapeutic ingredients for different health ailments [1]. The data are abundant enough to justify medicinal herbs as an alternate to, and future of, conventional and/or synthetic antimicrobials. Likewise, evidence validating the adverse health effects of synthetic food additives, and their injudicious application in the food industry, warrants exploring an extended role for natural ingredients in food processing and preservation. Growing evidence on the risks of health hazards linked to injudicious application of synthetic food additives is suggesting limiting their application and exploring relatively safer choices of natural origin [2,3]. During the last few decades, polyphenols have gained considerable attention as promising additives for improving food quality and the potential to prevent the risk of certain diseases, including oxidative stress, inflammation, and cancer [4].

A famous culinary herb in the *Lamiaceae* family, *Ocimum basilicum* L., known as sweet basil, is native to India and other Asian countries such as Pakistan, but it is now grown all over the world [5]. As a folk cure, basil leaves have long been used to treat a wide range of health issues. These include cancer, tremors, dysentery, mental disorders, inflammation, biliousness, tooth decay, and bronchitis [6]. The traditional claims are well supported by pharmacological evidence, including radical scavenging, anticancer, antipain, anti-infective, and immunomodulatory properties [7]. These bioactivities are ascribed to phenolic acids, flavonoids, rosmarinic acid, aromatic compounds, and *O. basilicum* essential oils such as eugenol, chavicol, linalool, and α-terpineol [8,9]. Ethanol/water extracts of *O. basilicum* leaves exhibit considerable antioxidant potential and antibacterial activity against Gram-positive and Gram-negative bacteria [10]. Recent evidence also reports that leaf extracts of *O. basilicum* possess anti-inflammatory, neuroprotective, immunoprotective, antidiabetic, cardioprotective, antistress and antitussive properties [11,12,13,14,15]. *O. basilicum* leaves were also reported to possess antimelanoma and radioprotective characteristics toward metastatic melanoma cell lines [16]. Quercetin and rutin have been listed as the most common flavonoids of sweet basil and possess anti-inflammatory and cytoprotective characteristics towards different cancers [17,18].

Common culinary applications of *O. basilicum* have been cited and are not limited to seasoning of soups, tomato-based dishes, spinach, sandwiches, cream cheese, dips, pasta, and a variety of squashes [19], but the herb also offers shelf-life stability properties for nutrient dense perishable goods [20]. Recently, bread prepared with *O. basilicum* extracts was reported to offer better antioxidant activity than breads incorporated with ascorbic acid and potassium sorbate [10]. *O. basilicum*, as a culinary herb of universal acceptance, has been substantially explored in terms of phytochemical composition, yet the variability in chemical composition of various anatomical fractions of the plant and their biological potential is still suggested to be mapped to different geographical settings. The aromatic profile of basil, characterized by the quality of its essential oil, is markedly influenced by the vegetative stage of the plant [19]. In Italy, small and young leaves of a basil plant that is 10–12 cm height are considered to be most relishing for pesto production [19,21]. Studies suggest basil plants at preferred growth stages possess higher concentrations of methyleugenol, which is considered toxic due to its structural resemblance to carcinogens such as estragole and safrole [22,23,24,25]. The current study was therefore planned to determine the link between growth stages and variability in toxicological response for the leaf extracts of *O. basilicum* cultivated in the temperate climate of Southern Punjab, Pakistan. The GS with the least toxicity or a non-toxic response was analyzed further for phytochemical composition, and antioxidant properties.

## 2. Materials and Methods

### 2.1. Collection of Plant Material

Sweet basil seeds were procured from a local market in Multan and verified by the botanist at Bahauddin Zakariya University, Multan, Pakistan. Voucher samples of the *O. basilicum* seeds were stored for future reference at the Institute of Food Science and Nutrition. The seeds were sown at a mean temperature and relative humidity of 27.2% and 34 °C, respectively, in a green house facility at the Faculty of Agricultural Sciences and Technology on 17 March 2019. The leaves of the plant were harvested at three growth stages, viz., GS-1 (15 cm plant height, 6 weeks post germination), GS-2 (31 cm plant height, 8 weeks post-germination), and GS-3 (61 cm plant height, 11 weeks post germination). Approximately 200 g of basil leaves (~20 leaves per plant) was collected for experimentation. Leaves collected from each stage were washed with distilled water, shade-dried, and stored in air-tight plastic containers at 4 °C for further analysis.

### 2.2. Chemicals and Equipment

Solvents for extraction (*n*-hexane, dichloromethane, ethanol) as well as reference and reagents for antioxidant and cytotoxicity analysis (tannic acid, gallic acid, ferrous sulphate, quercetin, Folin–Denis reagent, 2,4,6-tripyridyl-s-triazine (TPTZ), etoposide) were purchased from the local Sigma-Aldrich distributor. The chemicals, reagents and solvents used in this study were of analytical grade unless otherwise stated. Equipment used in analyses were: rotary evaporator (Hei-VAP, Heidolph, Schwabach, Germany), UV-Vis spectrophotometer (UV-Vis 3000, Dresden, Germany), and LC-ESI-MS/MS (LTQ XL, Thermo Electron Corporation, Walthan, MA, USA).

### 2.3. Preparation of O. basilicum Extracts

Non-sequential extraction of *O. basilicum* was performed with *n*-hexane, dichloromethane, ethanol, and water. Dehydrated leaves were ground to a mesh size below 80 mm using a kitchen grinder. Powdered leaves were subsequently extracted by suspending 20 g powder in 200 mL solvent. Extraction was performed using a rotary shaker while extraction conditions were set as 100 rpm, 24 h and 35 °C. Homogenates were filtered through Whatman filter paper no. 1. The filtrates recovered were condensed by rotary evaporation at 40 °C and freeze-dried to powder. Dried extracts were further used in bioassays after dissolution in respective solvents.

### 2.4. Brine Shrimp Lethality Test

Brine shrimp lethality assay was performed in accordance with the method followed by Ayaz et al. [26]. Artificial seawater was prepared by dissolving commercial salt mixture in distilled water. Fifty milligrams of shrimp (*Artemia salina*) eggs provided by Husein Ebrahim Jamal Research Institute of Chemistry (HEJ), Karachi, Pakistan, were sprinkled into the artificial seawater in 22 cm × 32 cm dark compartment of a rectangular plastic tray. Incubation was performed for 48 h at 37 °C and the hatched larvae were collected with Pasteur pipettes. Aqueous extracts of *O. basilicum* leaves at three different growth stages were prepared at concentrations of 10, 100, and 1000 µg/mL. Samples with different strengths were individually transferred to clean vials. Incubation vials were prepared by transferring 1 mL of artificial sea water and 30 shrimps into each vial. Final volume of the vial was filled to 5 mL with artificial seawater and pH of the shrimp growth medium was adjusted to 7.4 using 1N NaOH. The vials were incubated for 24 h at 26 °C. Survival rate of the shrimps in all vials including positive control, i.e., etoposide, was counted.

### 2.5. Preparation of O. basilicum Leaf Extracts Using Different Solvents

Basil leaf powder which showed minimum toxicity at 1000 µg/mL was subjected to extraction for further analysis. Extraction was performed with different solvents: *n-*hexane, dichloromethane, ethanol, and water in accordance with the method described in Section 2.3. Extracts recovered were stored at −18 °C and phytochemical qualitative screening, phytochemical quantification analysis, and antioxidant potential were evaluated. 

### 2.6. Phytochemical Analysis of O. basilicum Leaf Extracts

#### 2.6.1. Qualitative Analysis of Phytochemicals

Screening of the *O. basilicum* leaf extracts was performed for phenols, alkaloids, tannins, flavonoids, steroids, terpenoids, and saponins in accordance with the procedure of Kokate et al. [27] briefly presented in Table 1.

#### 2.6.2. Determination of Total Phenolic and Total Flavonoid Content

Total phenolic content in *O. basilicum* leaf extracts was measured using Folin–Ciocalteu reagent method previously adopted by Hossain and Shah, (2015) [28] using gallic acid as standard. Initially, 0.5 mL of *O. basilicum* extract was added to the test tube containing 1.2 mL Na_2_CO_3_ and 1.5 mL Folin–Ciocalteu reagent. Tubes were incubated in darkness for 30 min and absorbance was recorded at 765 nm via UV-Vis spectrophotometer. Ethanol was used as blank and results were presented as mg gallic acid equivalents (GAE)/g dried extract.

Aluminum chloride assay was used to determine the total flavonoid content in *O. basilicum* leaf extracts as adopted by Oriakhi et al. (2014) [29]. *O. basilicum* leaf extract (0.5 mL) was added to a test tube containing 0.5 mL distilled water, 0.15 mL NaNO_2_, and 0.15 mL of AlCl_3_. Contents of the tubes were incubated for 30 min in darkness and absorbance was read at 510 nm via UV-Vis spectrophotometer. Quercetin was used as standard, ethanol as blank, and results were expressed as mg quercetin equivalent (QE)/g dried extract.

Total tannin content in *O. basilicum* leaf extracts was measured by the Folin–Denis method [30] using tannic acid as standard. Absorbance values of the samples and standard were recorded at 725 nm using spectrophotometer. Total tannin content in *O. basilicum* leaf extracts was expressed as mg tannic acid equivalent (TAE)/100 g dried extract.

### 2.7. Determination of Radical Scavenging Activity

#### 2.7.1. DPPH Radical Scavenging Activity

DPPH free radical scavenging activity of the *O. basilicum* leaf extracts was determined by the method recommended by Alara et al. [31]. Initially, 1 mL of the *O. basilicum* leaf extracts (1 g/20 mL) was mixed with 3 mL of DPPH solution (0.004%) prepared in methanol. After 30 min incubation, the absorbance was recorded at 517 nm. Quercetin (125 µg/mL) was used as positive control (standard), while distilled water was used as blank (control). The DPPH inhibition percentage was computed from the below mentioned equation and results were expressed as % inhibition.

%Inhibtion=[(abs of control−abs of sample)÷abs of control]×100

where abs of control is the absorbance of DPPH radicals + distilled water and abs sample is absorbance of DPPH radicals + sample extracts/standard.

#### 2.7.2. Ferric Reducing Antioxidant Power (FRAP)

FRAP assay was conducted according to Zahin et al. [32]. FRAP working solution was prepared by mixing 10 mmol/L TPTZ, 20 mmol/L FeCl_3_, 300 mmol/L acetate buffer (pH 3.6) in a ratio of 1:1:10 (*v*/*v*/*v*). A test sample of 100 µL (1 g/20 mL) was mixed with FRAP working solution. Absorbance was recorded at 593 nm after 10 min reaction time at 37 °C. Ferrous sulphate was used as reference sample and outcomes were expressed as Fe µmol/g dried extract.

#### 2.7.3. Hydrogen Peroxide Scavenging Activity

Hydrogen peroxide scavenging activity of *O. basilicum* extracts was analyzed according to Ruch et al. [33]. A 40 mM H_2_O_2_ solution was prepared with 50 mM phosphate buffer (pH 7.4). The 100 µL test samples (1 g/20 mL) and 0.6 mL H_2_O_2_ solution were mixed, and incubated for 10 min. The absorbance of the samples was recorded at 230 nm on a spectrophotometer. Phosphate buffer solution was used as control and quercetin as standard. *O. basilicum* extract H_2_O_2_ scavenging activity was calculated as follows.

$$H_{2}O_{2}\;\mathrm{scavenging}\;\mathrm{activity}\;\left(\%\right)=[\left(abs\;of\;control-abs\;of\;sample\right)÷abs\;of\;control]\times 100$$

where abs of control is the absorbance of H_2_O_2_ + phosphate buffer and abs sample is absorbance of H_2_O_2_ + sample extract/standard.

### 2.8. Characterization of Phenolic Compounds by LC-ESI-MS-MS

To verify strong antioxidant potential, extracts were subjected to bioactive profiling through LC-ESI-MS/MS analysis [34]. Detection was performed by employing direct injection mode ESI (electron spray ionization) in both positive and negative modes. The mass range, capillary temperature, and flow rate was kept as *m*/*z* 50 to 1000, 280 °C, and 8 μL/min, respectively. Collision-induced energy generated during MS/MS analysis depended upon the nature/type of parent molecular ion subjected to 10 to 45 eV. For better ionization and ion transfer, each compound was optimized for MS parameters. Similarly, parent and daughter signals were also optimized either manually or by analyte infusion, while source parameters remained the same for each analyte. The structures of the compounds were identified using online software and compared with published literature (www.chemspider.com, accessed on 24 August 2021).

### 2.9. Statistical Analysis

The data presented are expressed as mean ± standard deviation of three replicates. The data were subjected to one-way and two-way analysis of variance (factorial ANOVA) where applicable, and the differences between the sample means were determined with the least significance difference test using Minitab (V17, LLC). A *p*-value ≤ 0.05 was considered as significant.

## 3. Results and Discussion

### 3.1. Cytotoxicity of O. basilicum Water Extracts

The brine shrimp lethality assay is a cost effective, quick, and reliable method of performing toxicological screening of herbal medicines and plant extracts [35]. In the present study, *O. basilicum* leaf aqueous extracts from three growth stages (GS-1, GS-2, GS-3) were examined for their possible cytotoxicity on brine shrimps using different concentrations. The results revealed that mortality percentage of aqueous extracts on brine shrimps was notably influenced by the growth stages. Whereas dose-dependent mortality was observed for GS-1 and GS-3, this was not the case for GS-2 (Table 2).

In addition, basil crude extracts when tested at three different concentrations (10, 100, and 1000 µg/mL) were recorded as producing non-substantial toxicological effects. Lowest toxicity as identified from the brine shrimp mortality index was observed at the second growth stage of *O. basilicum* (Table 2).

The mortality rate at GS-1, GS-2, and GS-3 was observed as 26.7 ± 3.34%, 8.91 ± 0.10%, and 16.7 ± 0.34% at 1000 µg/mL. Interestingly, the highest mortality of brine shrimps exposed to *O. basilicum* leaf aqueous extracts (GS-1 and GS-3) was observed at 1000 µg/mL while the lowest mortality rate was recorded at 10 µg/mL exposure levels. In a previous study, ethanol extracts of *O. basilicum* leaves collected from Sudan were recorded as having LC_50_ greater than 1000 µg/mL in the brine shrimp assay [36], which supports the findings of the present study. The recent findings, on the other hand, contrast with those of Khan et al. [37] wherein *O. basilicum* leaf crude extracts showed LC_50_ value of 91.6 µg/mL in the brine shrimp lethality assay. It has been reported that toxicological effects of *O. basilicum* vary depending on the cultivar, plant age and height, biochemical content, extract type, plant part studied, and chemotype [21].

Relying on the results obtained using the brine shrimp test, ethanolic basil leaf extract recovered from *O. basilicum* during the second growth stage was selected for further analysis due to less toxic effects.

### 3.2. Qualitative Phytochemical Screening of O. basilicum Extracts

The qualitative examination revealed the presence of both primary and secondary plant metabolites in various basil crude extracts (Table 3). Alkaloids, phenols, tannins, flavonoids, terpenoids, steroids, and glycosides were detected in basil leaf aqueous extracts but saponins were absent. Alkaloids were also absent in *n*-hexane extract; alkaloids, terpenoids, and glycosides in ethanol extract; and alkaloids, saponins, and terpenoids in dichloromethane extract. The results of Gebrehiwot et al. [38] and Hamad et al. [39] support the findings of the current investigation wherein chloroform/methanol (1:1, *v*/*v*) and aqueous extracts of *O. basilicum* leaves, respectively, were also detected with these phytochemicals during screening tests. Corroborating the recent findings, a previous study of aqueous extracts of *O. basilicum*, which are traditionally used in folklore medicine were reported to contain higher to trace amounts of alkaloids, saponin, flavonoids, steroids, terpenes, tannins, coumarins, and carbohydrates [40,41].

Our qualitative screening results for the *O. basilicum* crude extracts validate the presence of a wide range of phytochemicals thus indicating the plant’s ability to provide the anticipated promising biological response. Strong correlation has been established between the secondary plant metabolites of *Ocimum* spp. and their bioactivity including radical scavenging activity in a study by Das et al. [42]. In another study by Daniel et al. [43], the secondary plant metabolites bearing promising radical scavenging activity were suggested to improve healing processes. Crude extracts, as have been deployed in this study, are considered as a pool of bioactive compounds. While polarity of the solvent is the key concern in extracting secondary plant metabolites, the said property had also been reported to influence antioxidant and toxicological activities of the crude extracts. In a surge of food-grade plant extracts, though aqueous leaf extracts of *O. basilicum* yield a range of secondary metabolites few of these (such as tannins and cyanogens) may have toxicological effects for the consumer [44]. Contrary to the above, aqueous extracts of *O. basilicum* were not found to limit toxicological risk for consumers.

### 3.3. Total Phenolic Content, Total Flavonoid Content, and Tannin Content of O. basilicum Extracts

**Figure 1:**
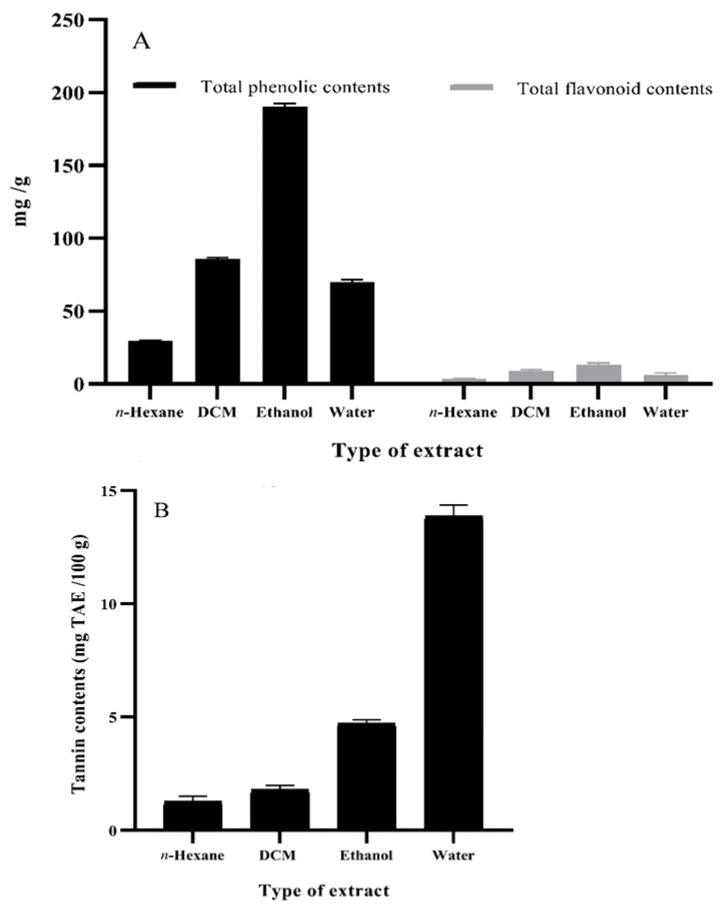
Phytochemical content of different solvent extracts of basil (*O. basilicum*; 2.5 mg/mL). (**A**) Total phenolic (mg GAE/g) and total flavonoid content (mg QE/g). (**B**) Tannin content (mg TAE/100 g). Values are means ± S.D. GAE, gallic acid equivalent; QE, quercetin equivalent; TAE, tannic acid equivalent; DCM, dichloromethane.

In the present study, ethanol extracts of *O. basilicum* leaves were recorded to have more affinity towards the extraction of total phenolic (191.2 mg GAE)/g content, considerably more than identified in dichloromethane (86.6 mg GAE/g) and aqueous (70.7 mg GAE/g) extracts. In contrast, *n*-hexane extracts of *O. basilicum* leaves exhibited the lowest potential of total phenolic content at 29.7 mg GAE/g (Figure 1A). The current findings are consistent with those of Vlase et al. [45], who found 175.5 mg GAE/g of total phenolic content in *O. basilicum* ethanol extract. Another study reported 45.4 mg GAE/g total phenolic content in 80% aqueous methanol extracts of *O. basilicum* [46]. Likewise, aqueous extracts of *O. basilicum* were reported to hold comparable total phenolic content, i.e., 96.4 mg GAE/g [39], that corroborates the findings of our work. Previously, the findings of Kim et al. [47] and Shiga et al. [48] corelated the radical scavenging capacity of basil leaf extracts with total phenolic content. Our study also reported on rosmarinic acid, an important phenolic compound in *O. basilicum* that exhibits substantial radical scavenging, antipain, antiviral, and antimicrobial activities.

Ethanol extracts of *O. basilicum* leaves were noted to contain higher total flavonoid, i.e., 13.3 mg, and quercetin equivalent (QE)/g) content than dichloromethane (9.63 mg QE/g) and aqueous (6.49 mg QE/g) extracts. In contrast, the least affinity towards extraction of total flavonoids was shown by *n*-hexane extracts, i.e., 3.51 mg QE/g (Figure 1A). Vlase et al. [45] reported notable concentrations of total flavonoid content (6.72 ± 0.19 mg rutin equivalent (RE)/g) in ethanol extracts of *O. basilicum*. Similarly, Hamad et al. [39] observed 32.7 mg catechol/g of total flavonoid content in aqueous extracts of *O. basilicum* which substantiates the results of the current study.

Higher concentrations of total tannins (13.3 g mg tannic acid equivalent (TAE)/100 g) were observed in *O. basilicum* leaf aqueous extract than ethanol (4.60 mg TAE/100 g), dichloromethane (1.90 mg TAE/100 g), and *n*-hexane (1.20 mg TAE/100 g) extracts (Figure 1B). Akin to the present investigation, in previous studies polar solvent extracts, viz., methanol, ethanol, and ethyl acetate, of *O. basilicum* leaves were reported with total tannin content, i.e., 54.2, 56.0, and 46.5 mg, catechin equivalent (CE)/g dry extract, respectively, which is notably higher than 4.49 mg CE/g reported for dichloromethane extracts [49]. Moreover, Tewari et al. [50] also found *O. basilicum* leaf extracts rich in total tannin content (2.32 ± 0.56 mg TAE/100 g).

### 3.4. Antioxidant Potential of O. basilicum Extracts

**Figure 2:**
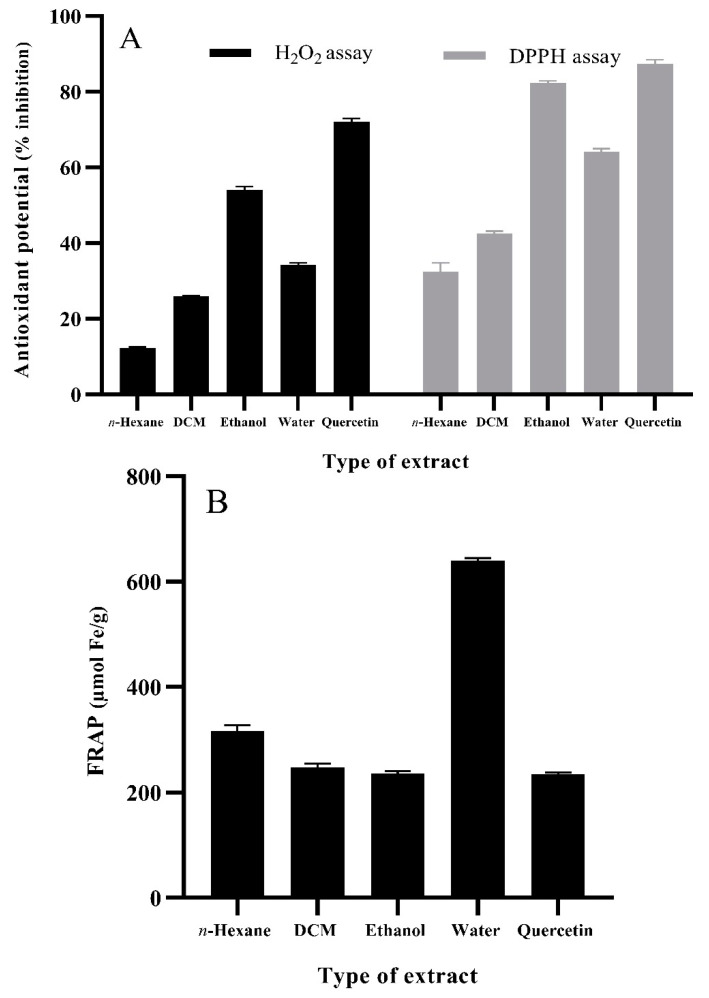
Antioxidant potential of different solvent extracts of basil (*O. basilicum*: 50 mg/mL; positive control quercetin: 125 µg/mL): (**A**) H_2_O_2_ and DPPH assay; (**B**) FRAP assay. Values are means ± S.D. DCM, dichloromethane.

Antioxidant potential of plant extracts can be evaluated by employing widely accepted techniques, i.e., stable free radial scavenging using the DPPH assay, ferric reducing antioxidant power (FRAP), and scavenging of hydrogen peroxide (H_2_O_2_) [51]. In our study, ethanolic extracts of *O. basilicum* leaves outlined notable antioxidant activity in DPPH (82.4%), H_2_O_2_ (54.0%) (Figure 2A), and FRAP (237 µmol Fe/g) (Figure 2B) assays. In addition, aqueous and dichloromethane extracts presented moderate antioxidant activity, whereas *n*-hexane extracts bared non-influential results in all three assays, i.e., DPPH (32.4%), FRAP (316 µmol Fe/g), and H_2_O_2_ (12%). It can be concluded from the findings of the antioxidant assays that the higher radical scavenging activity of ethanolic *O. basilicum* leaf extracts may be attributed to the presence of phenolics, flavonoids, and tannins. The findings of Ahmed et al. [52] also outlined a positive relationship among antioxidant activity, phenolic content, and geographical location. It was also observed that 70% ethanol extract of *O. basilicum* (Minia location) offered higher phenolic content (82.45 mg pyrocatechol equivalents (PE)/g) and antioxidant activity determined via DPPH assay (IC_50_ 1.29 mg/mL) when compared with *O. basilicum* extracts from two other locations, i.e., Assiut and Beni Suef. The antioxidant potential of *O. basilicum* leaves was also reported by Mahirah et al. [53] suggesting methanolic extracts of freeze-dried leaves offer the highest DPPH scavenging activity, i.e., 92.6%, followed by ethanolic extracts (42.6%), and aqueous extract. Sekar et al. [54] also reported *O. basilicum* leaves to hold substantial antioxidant potential as determined by DPPH assay.

### 3.5. LC-ESI-MS/MS Phenolic Compound Identification

The ESI-MS/MS data exhibited the presence of liquiritigenin (flavanone), catechin (flavan-3-ol), umbelliferone (hydroxycoumarin), ellagic acid, and rosmarinic acid (phenolic acid) in the ethanol extract as shown in Table 4. Umbelliferone and liquiritigenin were identified as novel compounds from the ethanolic extracts of *O. basilicum* leaves. There have been previous reports suggesting *O. basilicum* leaves contain rosmarinic acid, a bioactive compound known for its significant radical scavenging and antibacterial properties [47,48]. Basil has also been reported to hold catechin and ellagic acid, already acknowledged for their antioxidant activities [55,56]. Liquiritigenin isolated from *Glycyrrhizae radix* has been shown to protect osteoblasts from oxidative stress and mitochondrial dysfunction in MC3T3-E1 cells through its antioxidant properties [57]. Liquiritigenin has also been reported as having notable radical scavenging and antimicrobial activities, as demonstrated by Ramalingam et al. [58]. Umbelliferone, a phenolic compound identified in the present study, has also been reported as a significant free radical scavenging compound [59].

## 4. Conclusions

The current study was conducted in order to assess the toxicity, phytochemicals, antioxidant potential, and bioactive profiling of basil leaves at three different growth stages. The results of the current research showed that basil leaf extracts did not show potential toxicity and are an excellent source of natural antioxidants. The presence of phenolic compounds is associated with strong antioxidant potential. Furthermore, LC-ESI-MS/MS profiling revealed the presence of compounds having strong antioxidant potential including rosmarinic acid, ellagic acid, catechin, liquiritigenin, and umbelliferone. These findings support the utilization of basil as an additive in novel foodstuffs. Further extensive experimental research is also needed to validate the bioactive compound characterizations to be used as a natural preservative in food products.

## Figures and Tables

**Table 1:** Qualitative phytochemical screening methods for *O. basilicum* leaf extracts.

Test	Method	Observation	Constituents
Wagner’s Test	Extract (2 mL; 2 g/20 mL) + 1% HCL (1 mL) + Wagner’s reagent (0.5 mL)	Cream, reddish brown precipitate	Alkaloids
Ferric Chloride Test	Extract (2 mL; 0.5 g/5 mL) + 10% FeCl_3_ (few drops)	Dark brown or blackish red color	Flavonoids
Folin–Ciocalteu reagent Test	Extract (1 mL; 0.5 g/5 mL) + Folin–Ciocalteu reagent (0.5 mL) + aqueous sodium (few drops)	Gray or black color	Phenols
Froth Test	Extract (1 mL; 0.5 g/5 mL) + water (5 mL)	Copious lather formation	Saponins
Salkowki’s Test	Extract (1 mL; 0.2 g/2 mL) + chloroform (1 mL) + conc. H_2_SO_4_ (few drops)	Brown ring formation	Steroids
Ferric Chloride Test	Extract (1 mL; 0.5 g/10 mL) + 5% FeCl_3_ (3 drops)	Greenish brown, blue green or blue-black color	Tannins
Salkowki’s Test	Extract (2 mL; 0.5 g/5 mL) + chloroform (1 mL) + conc. H_2_SO_4_ (2 mL)	Red brownish precipitate	Terpenoids

**Table 2:** Brine shrimp lethality assay for *O. basilicum* leaf aqueous extracts.

Growth Stage (GS)	Percentage of Mortality at Various Concentrations of Extract		
	10 µg/mL	100 µg/mL	1000 µg/mL
GS-1	6.66 ± 0.02	10.0 ± 1.80	26.7 ± 3.34
GS-2	6.66 ± 0.03	6.66 ± 0.34	8.91 ± 0.10
GS-3	13.3 ± 0.33	13.3 ± 0.67	16.7 ± 0.34
Etoposide (standard)	73.2 ± 0.21	100 ± 0.01	100 ± 0.01

All results are presented as mean ± SD. GS-1, 58 days of growth period; GS-2, 69 days of growth period; GS-3, 93 days of growth period.

**Table 3:** Qualitative phytochemical screening of various *O. basilicum* leaf extracts.

Extract	Alkaloids	Flavonoids	Phenols	Steroid	Saponins	Tannins	Terpenoids	Glycosides
*n*-Hexane	−	+	+	+	+	+	+	+
Ethanol	−	+	+	+	+	+	−	−
Water	+	+	+	+	−	+	+	+
Dichloromethane	−	+	+	+	−	+	−	+

+; detected, −; not detected.

**Table 4:** LC-ESI-MS/MS main phenolic compound identification in ethanol extract of *O. basilicum*.

Average Mass (*m*/*z*)	Rt. (min)	ESI-MS/MS (Positive Mode)	Identification	Molecular Formula	References
256	4.21	255, 237	Liquiritigenin	C_15_H_12_O_4_	[59]
301	5.06	301, 286.08, 283, 258, 231.17, 186	Ellagic acid	C_14_H_6_O_8_	[60]
359	2.30	359.33, 344.08, 331.17, 315, 229.08, 197, 161	Rosmarinic acid	C_18_H_16_O_8_	[61]
290	12.55	291.25, 273, 247.25	Catechin	C_15_H_14_O_6_	[62]
161	1.27	161.08, 133	Umbelliferone	C_9_H_6_O_3_	[63]

## Data Availability

Available data are presented in the manuscript.

## References

[1] Ranjha M.M.A.N., Amjad S., Ashraf S., Khawar L., Safdar M.N., Jabbar S., Nadeem M., Mahmood S., Murtaza M.A. (2020). Extraction of polyphenols from apple and pomegranate peels employing different extraction techniques for the development of functional date bars. Int. J. Fruit Sci..

[2] Ribeiro B.G., Guerra J.M., Sarubbo L.A. (2020). Biosurfactants: Production and application prospects in the food industry. Biotechnol. Progress..

[3] Tortosa V., Pietropaolo V., Brandi V., Macari G., Pasquadibisceglie A., Polticelli F. (2020). Computational methods for the identification of molecular targets of toxic food additives. Butylated hydroxytoluene as a case study. Molecules.

[4] Efenberger-Szmechtyk M., Nowak A., Czyzowska A. (2021). Plant extracts rich in polyphenols: Antibacterial agents and natural preservatives for meat and meat products. Crit. Rev. Food Sci. Nutr..

[5] Rubab S., Hussain I., Khan B.A., Unar A.A., Abbas K.A., Khichi Z.H., Khan H. (2017). Biomedical Description of *Ocimum basilicum* L.. J. Islamic Int. Med. Coll..

[6] Janick J., Simon J.E. (1990). Advances in New Crops.

[7] Ch M.A., Naz S.B., Sharif A., Akram M., Saeed M.A. (2015). Biological and pharmacological properties of the sweet basil (*Ocimum basilicum*). J. Pharm. Res. Int..

[8] Purushothaman B., Srinivasan R.P., Suganthi P., Ranganathan B., Gimbun J., Shanmugam K. (2018). A comprehensive review on *Ocimum basilicum*. J. Nat. Remedies.

[9] Majdi C., Pereira C., Dias M.I., Calhelha R.C., Alves M.J., Rhourri-Frih B., Charrouf Z., Barros L., Amaral J.S., Ferreira I.C. (2020). Phytochemical characterization and bioactive properties of cinnamon basil (*Ocimum basilicum* cv.‘Cinnamon’) and lemon basil (*Ocimum× citriodorum*). Antioxidants.

[10] Takwa S., Caleja C., Barreira J.C., Soković M., Achour L., Barros L., Ferreira I.C. (2018). *Arbutus unedo* L. and *Ocimum basilicum* L. as sources of natural preservatives for food industry: A case study using loaf bread. LWT-Food Sci. Technol..

[11] Eftekhar N., Moghimi A., Mohammadian R.N., Saadat S., Boskabady M. (2019). Immunomodulatory and anti-inflammatory effects of hydro-ethanolic extract of *Ocimum basilicum* leaves and its effect on lung pathological changes in an ovalbumin-induced rat model of asthma. BMC Complement. Altern. Med..

[12] Kheradmandpour M., Aminifar S.A., Dianat M. (2020). The effect of hydro-alcoholic extract of *Ocimum basilicum* on CaCl_2_-induced cardiac Arrhythmias in rats. Jentashapir J. Cell Mol. Biol..

[13] Al-Subhi L. (2020). Two cultivars of *Ocimum basilicum* leaves extracts attenuate streptozotocin-mediated oxidative stress in diabetic Rrats. Pak. J. Biol. Sci..

[14] Shahrajabian M.H., Sun W., Cheng Q. (2020). Chemical components and pharmacological benefits of Basil (*Ocimum basilicum*): A review. Int. J. Food Prop..

[15] Seyed M.A., Ayesha S., Azmi N., Al-Rabae F.M., Al-Alawy A.I., Al-Zahrani O.R., Hawsawi Y. (2021). The neuroprotective attribution of *Ocimum basilicum*: A review on the prevention and management of neurodegenerative disorders. Future J. Pharm. Sci..

[16] Monga J., Sharma M., Tailor N., Ganesh N. (2011). Antimelanoma and radioprotective activity of alcoholic aqueous extract of different species of *Ocimum* in C57BL mice. Pharm. Biol..

[17] Saraf A., Sankhala S. (2014). Simultaneous determination of rutin and quercetin in different parts of *Tecomella undulata* (seem): An endangered medicinal plant. Int. J. Pharmacogn. Phytochem. Res..

[18] Ghasemzadeh A., Ashkani S., Baghdadi A., Pazoki A., Jaafar H.Z., Rahmat A. (2016). Improvement in flavonoids and phenolic acids production and pharmaceutical quality of sweet basil (*Ocimum basilicum* L.) by ultraviolet-B irradiation. Molecules.

[19] Miele M., Dondero R., Ciarallo G., Mazzei M. (2021). Methyleugenol in *Ocimum basilicum* L. Cv. genovese gigante. J. Agric. Food Chem..

[20] Zahran E.M., Abdelmohsen U.R., Khalil H.E., Desoukey S.Y., Fouad M.A., Kamel M.S. (2020). Diversity, phytochemical and medicinal potential of the genus *Ocimum* L. (Lamiaceae). Phytochem. Rev..

[21] Sestili P., Ismail T., Calcabrini C., Guescini M., Catanzaro E., Turrini E., Fimognari C. (2018). The potential effects of *Ocimum basilicum* on health: A review of pharmacological and toxicological studies. Expert Opin. Drug Metab. Toxicol..

[22] Touiss I., Khatib S., Bekkouch O., Amrani S., Harnafi H. (2017). Phenolic extract from *Ocimum basilicum* restores lipid metabolism in Triton WR-1339-induced hyperlipidemic mice and prevents lipoprotein-rich plasma oxidation. Food Sci. Hum. Wellness.

[23] Samson J., Sheeladevi R., Ravindran R. (2007). Oxidative stress in brain and antioxidant activity of *Ocimum sanctum* in noise exposure. Neurotoxicology.

[24] Hussain A.I., Anwar F., Sherazi S.T.H., Przybylski R. (2008). Chemical composition, antioxidant and antimicrobial activities of basil (*Ocimum basilicum*) essential oils depends on seasonal variations. Food Chem..

[25] Anwar F., Hussain A.I., Sherazi S.T.H., Bhanger M.I. (2009). Changes in composition and antioxidant and antimicrobial activities of essential oil of fennel (*Foeniculum vulgare* Mill.) fruit at different stages of maturity. J. Herbs Spices Med. Plants.

[26] Ayaz M., Junaid M., Ullah F., Sadiq A., Subhan F., Khan M.A., Ahmad W., Ali G., Imran M., Ahmad S. (2016). Molecularly characterized solvent extracts and saponins from *Polygonum hydropiper* L. show high anti-angiogenic, anti-tumor, brine shrimp, and fibroblast NIH/3T3 cell line cytotoxicity. Front. Pharmacol..

[27] Kokate C., Purohit A., Gokhale S. (2001). Carbohydrate and derived Products, drugs containing glycosides, drugs containing tannins, lipids and protein alkaloids. Text Book of Pharmacognosy.

[28] Hossain M.A., Shah M.D. (2015). A study on the total phenols content and antioxidant activity of essential oil and different solvent extracts of endemic plant *Merremia borneensis*. Arab. J. Chem..

[29] Oriakhi K., Oikeh E.I., Ezeugwu N., Anoliefo O., Aguebor O., Omoregie E.S. (2014). Comparative antioxidant activities of extracts of *Vernonia amygdalina* and *Ocimum gratissimum* leaves. J. Agric. Sci..

[30] Polshettiwar S.A., Ganjiwale R.O., Wadher S.J., Yeole P.G. (2007). Spectrophotometric estimation of total tannins in some ayurvedic eye drops. Indian J. Pharma. Sci..

[31] Alara O.R., Abdurahman N.H., Mudalip S.A., Olalere O.A. (2019). Effect of drying methods on the free radicals scavenging activity of *Vernonia amygdalina* growing in Malaysia. J. King Saud Univ. Sci..

[32] Zahin M., Aqil F., Ahmad I. (2010). Broad spectrum antimutagenic activity of antioxidant active fraction of *Punica granatum* L. peel extracts. Mutat. Res. Genet. Toxicol. Environ. Mutagen.

[33] Ruch R.J., Cheng S.J., Klaunig J.E. (1989). Prevention of cytotoxicity and inhibition of intercellular communication by antioxidant catechins isolated from Chinese green tea. Carcinogenesis.

[34] Steinmann D., Ganzera M. (2011). Recent advances on HPLC/MS in medicinal plant, analysis. J. Pharm. Biomed. Anal..

[35] Ramachandran S., Vamsikrishna M., Gowthami K.V., Heera B., Dhanaraju M.D. (2011). Assessment of cytotoxic activity of *Agave cantula* using Brine Shrimp (*Artemia salina*) lethality assay. Asian J. Sci. Res..

[36] Gadir S.A. (2012). Assessment of bioactivity of some Sudanese medicinal plants using brine shrimp (*Artemia salina*) lethality assay. J. Chem. Pharm. Res..

[37] Khan I., Ahmad K., Khalil A.T., Khan J., Khan Y.A., Saqib M.S., Umar M.N., Ahmad H. (2015). Evaluation of antileishmanial, antibacterial and brine shrimp cytotoxic potential of crude methanolic extract of Herb *Ocimum basilicum* (Lamacea). World J. Tradit. Chin. Med..

[38] Gebrehiwot H., Bachetti R.K., Dekebo A. (2015). Chemical composition and antimicrobial activities of leaves of sweet basil (*Ocimum basilicum* L.) herb. Int. J. Basic Clin. Pharmacol..

[39] Hamad G.M., Darwish A.M., Abu-Serie M.M., El Sohaimy S.A. (2017). Antimicrobial, antioxidant and anti-inflammatory characteristics of combination (*Cassia fistula* and *Ocimum basilicum*) extract as natural preservative to control & prevent food contamination. J. Food Nutr. Res..

[40] Sanni S., Onyeyili P.A., Sanni F.S. (2008). Phytochemical analysis, elemental determination and some in vitro antibacterial activity of *Ocimum basilicum* L. leaf extracts. Res. J. Phytochem..

[41] Azam M., Irshad S. (2016). Phytochemical screening and antibacterial activities of essential oil, ethanolic and methanolic extracts of *Ocimum basillicum* L.. Pak. J. Biochem. Mol. Biol..

[42] Das S., Barman S., Teron R., Bhattacharya S.S., Kim K.H. (2020). Secondary metabolites and anti-microbial/anti-oxidant profiles in *Ocimum* spp.: Role of soil physico-chemical characteristics as eliciting factors. Environ. Res..

[43] Daniel V.N., Daniang I.E., Nimyel N.D. (2011). Phytochemical analysis and mineral elements composition of *Ocimum basilicum* obtained in JOS metropolis, plateau state, Nigeria. Int. J. Eng. Sci. Technol..

[44] Nguyen V.T., Nguyen N.Q., Thi N.Q.N., Thi C.Q.N., Truc T.T., Nghi P.T.B. Studies on chemical, polyphenol content, flavonoid content, and antioxidant activity of sweet basil leaves (*Ocimum basilicum* L.). Proceedings of the IOP Conference Serie Materials Science and Engineerings, 2nd International Conference on Innovative Technology.

[45] Vlase L., Benedec D., Hanganu D., Damian G., Csillag I., Sevastre B., Mot A.C., Silaghi-Dumitrescu R., Tilea I. (2014). Evaluation of antioxidant and antimicrobial activities and phenolic profile for *Hyssopus officinalis, Ocimum basilicum* and *Teucrium chamaedrys*. Molecules.

[46] Naidu J.R., Ismail R.B., Sasidharan S. (2016). Chemical profiling and antioxidant activity of Thai basil (*Ocimum basilicum*). J. Essent. Oil-Bear. Plants..

[47] Kim H.J., Chen F., Wang X., Rajapakse N.C. (2006). Effect of methyl jasmonate on secondary metabolites of sweet basil (*Ocimum basilicum* L.). J. Agric. Food Chem..

[48] Shiga T., Shoji K., Shimada H., Hashida S.N., Goto F., Yoshihara T. (2009). Effect of light quality on rosmarinic acid content and antioxidant activity of sweet basil, *Ocimum basilicum* L.. Plant Biotechnol..

[49] Harnafi H., Caid H.S., el Houda Bouanani N., Aziz M., Amrani S. (2008). Hypolipemic activity of polyphenol-rich extracts from *Ocimum basilicum* in Triton WR-1339-induced hyperlipidemic mice. Food Chem..

[50] Tewari D., Pandey H.K., Sah A.N., Meena H., Chander V., Singh R., Singh P. (2015). Phytochemical, antioxidant and antidepressant evaluation of *Ocimum basilicum, O. tenuiflorum, O. kilimandscharicum* grown in India. J. Biol. Act. Prod. Nat..

[51] Lim C.S.H., Lim S.L. (2013). Ferric reducing capacity versus ferric reducing antioxidant power for measuring total antioxidant capacity. Lab. Med..

[52] Ahmed A.F., Attia F.A., Liu Z., Li C., Wei J., Kang W. (2019). Antioxidant activity and total phenolic content of essential oils and extracts of sweet basil (*Ocimum basilicum* L.) plants. Food Sci. Hum. Wellness.

[53] Siti Mahirah Y., Rabeta M.S., Antora R.A. (2018). Effects of different drying methods on the proximate composition and antioxidant activities of *Ocimum basilicum* leaves. Food Res..

[54] Sekar K., Thangaraj S., Babu S.S., Harisaranraj R., Suresh K. (2009). Phytochemical constituent and antioxidant activity of extract from the leaves of *Ocimum basilicum*. J. Phytol..

[55] Wang X., Tong H., Chen F., Gangemi J.D. (2010). Chemical characterization and antioxidant evaluation of muscadine grape pomace extract. Food Chem..

[56] Zhang H.M., Zhao L., Li H., Xu H., Chen W.W., Tao L. (2014). Research progress on the anticarcinogenic actions and mechanisms of ellagic acid. Cancer Biol. Med..

[57] Choi E.M., Suh K.S., Lee Y.S. (2014). Liquiritigenin restores osteoblast damage through regulating oxidative stress and mitochondrial dysfunction. Phytother Res..

[58] Ramalingam M., Kim H., Lee Y., Lee Y.I. (2018). Phytochemical and pharmacological role of liquiritigenin and isoliquiritigenin from radix glycyrrhizae in human health and disease models. Front. Aging Neurosci..

[59] Kaur P., Kumar M., Singh B., Kumar S., Kaur S. (2012). Amelioration of oxidative stress induced by oxidative mutagens and COX-2 inhibitory activity of umbelliferone isolated from *Glycyrrhiza glabra* L.. Asian Pac. J. Trop. Biomed..

[60] Rahman H., Khan I., Hussain A., Shahat A.A., Tawab A., Qasim M., Adnan M., Al-Said M.S., Ullah R., Khan S.N. (2018). *Glycyrrhiza glabra* HPLC fractions: Identification of aldehydo isoophiopogonone and liquirtigenin having activity against multidrug resistant bacteria. BMC Complement. Altern. Med..

[61] Yan L., Yin P., Ma C., Liu Y. (2014). Method development and validation for pharmacokinetic and tissue distributions of ellagic acid using ultrahigh performance liquid chromatography-tandem mass spectrometry (UPLC-MS/MS). Molecules.

[62] Chen H., Zhang Q., Wang X., Yang J., Wang Q. (2011). Qualitative analysis and simultaneous quantification of phenolic compounds in the aerial parts of *Salvia miltiorrhiza* by HPLC-DAD and ESI/MSn. Phytochem. Anal..

[63] Riaz M., Rasool N., Iqbal M. (2017). Liquid chromatography-electrospray ionization-tandem mass spectrometry (LC-ESI-MS/MS) analysis of *Russelia equisetiformis* extract. Bulg. Chem. Commun..

